# Impact of Cryopreservation on Motile Subpopulations and Tyrosine-Phosphorylated Regions of Ram Spermatozoa during Capacitating Conditions

**DOI:** 10.3390/biology10111213

**Published:** 2021-11-20

**Authors:** Patricia Peris-Frau, Irene Sánchez-Ajofrín, Alicia Martín Maestro, Carolina Maside, Daniela Alejandra Medina-Chávez, Olga García-Álvarez, María del Rocío Fernández-Santos, Vidal Montoro, José Julián Garde, Manuel Ramón, Ana Josefa Soler

**Affiliations:** 1 SaBio IREC (CSIC-UCLM-JCCM), ETSIAM, Campus Universitario s/n, 02071 Albacete, Spain; patricia.peris@uclm.es (P.P.-F.); irene.ssanchez@uclm.es (I.S.-A.); aliciaisabel.martinmaestro@alu.uclm.es (A.M.M.); carolina.maside@uclm.es (C.M.); daniela.medina@uclm.es (D.A.M.-C.); olga.garcia@uclm.es (O.G.-Á.); mrocio.fernandez@uclm.es (M.d.R.F.-S.); vidal.montoro@uclm.es (V.M.); julian.garde@uclm.es (J.J.G.); 2 CERSYRA-IRIAF, Av. del Vino, 10, 13300 Valdepeñas, Spain; m.ramon.fernandez@gmail.com

**Keywords:** computer-assisted sperm analysis, cytometry, subpopulations, sperm capacitation, sperm cryopreservation, tyrosine phosphorylation

## Abstract

**Simple Summary:**

Spermatozoa go through diverse changes to achieve their fertilizing potential (capacitation) and develop a specific motility pattern (hyperactivation). However, to ensure a greater reproductive success, not all the spermatozoa present in an ejaculate react equally or at the same time. Therefore, a comparative analysis was performed in the present study to improve our current understanding about how cryopreservation may affect the heterogeneous nature of fresh ejaculates during these two events. Among the four motile sperm subpopulations identified in fresh and frozen-thawed ram semen, one of them developed a hyperactivated-like movement and was the main group involve in those changes associated with sperm capacitation based on the marked increase and the positive correlation with mitochondrial activity and tyrosine phosphorylation, two relevant parameters that usually increase during capacitation. In addition, cryopreservation altered the distribution of the motile sperm subpopulations. Although the subpopulation with hyperactivated-like movement increased at the beginning of incubation in frozen-thawed samples, this subpopulation together with the subpopulation of rapid and progressive spermatozoa were replaced after a prolonged incubation by the subpopulation of slow spermatozoa with the lowest mitochondrial activity, which clearly indicate the reduction in sperm quality. These findings will aid to optimize the current cryopreservation and in vitro fertilization protocols.

**Abstract:**

The heterogeneous nature of ejaculates highlights the relevance of studying the behavior of different sperm subpopulations. Changes in sperm motility and the increase in tyrosine phosphorylation are key events that usually occur during capacitation and can be modified by the cryopreservation process. However, the relationship between both events remains poorly defined throughout capacitation in the different sperm subpopulations. Fresh and frozen-thawed spermatozoa were incubated in capacitating (CAP) and non-capacitating (NC) media up to 240 min. Sperm kinematics, tyrosine phosphorylation and mitochondrial activity were measured by the CASA system and imaging flow cytometry. Four motile sperm subpopulations (SP) were identified in fresh and frozen-thawed ram semen after the cluster analysis. Incubation under CAP conditions over time led to greater changes in the percentage of spermatozoa included in each subpopulation compared to NC conditions, being different between fresh and frozen-thawed spermatozoa. The SP1, characterized by slow spermatozoa, progressively increased after 15 min in frozen-thawed samples incubated in both media but not in fresh ones. The SP4, characterized by fast and non-linear spermatozoa, showed a marked increase during CAP, but not under NC conditions, occurring more rapidly in frozen-thawed spermatozoa. This subpopulation (SP4) was also the only one positively and strongly correlated with mitochondrial activity and all phosphorylated sperm regions during capacitation, either in fresh or frozen-thawed samples. Our results indicated that in vitro capacitation induced significant changes in the distribution of motile sperm subpopulations, being affected by cryopreservation. Notwithstanding, the subpopulation which probably represents hyperactivated-like spermatozoa (SP4) also increased in frozen-thawed samples, occurring faster and simultaneously to the increment of mitochondrial activity and tyrosine phosphorylation of different sperm regions.

## 1. Introduction

In most mammal species, the existence of heterogeneous subpopulations of spermatozoa in the ejaculates with distinct kinematic and morphometric characteristics has been widely documented [1,2,3,4,5,6]. Some authors have suggested that the fertilizing ability of fresh or frozen-thawed ejaculates could reside mainly in that sperm subpopulation with the fastest and progressive motility [7,8,9], because this type of movement seems necessary to get through the different parts of the female reproductive tract and reach the oviduct, where the physiological process called capacitation takes place. However, the motility pattern of spermatozoa shifts at some point during capacitation from rapid, progressive, and symmetrical flagellar beating towards a more vigorous, asymmetrical, and less progressive movement with higher amplitude of flagellar bend, which is widely known as hyperactivation [10,11].

Only hyperactivated spermatozoa can leave the oviductal reservoir, pass the intraluminal mucus and penetrate the extracellular matrix of oocytes [12,13]. However, the acquisition of this specific motility pattern is not the only requirement for fertilization. Spermatozoa must undergo an extensive remodeling during capacitation to achieve their fertilizing ability, which includes an increase in membrane fluidity, protein tyrosine phosphorylation, energy metabolism and mitochondrial activity, as well as changes in protein and calcium levels, actin polymerization, chromatin decondensation and hyperpolarization of the plasma membrane [6,14,15,16,17,18,19,20,21]. However, not all the sperm subpopulations present in an ejaculate have the same sensitivity to physiological or biotechnological processes such as capacitation or cryopreservation [1,22,23]. In mouse, only a sperm subpopulation showed membrane hyperpolarization during capacitating conditions [24]. Ramió-Lluch et al. [25] found that in vitro capacitation induced significant changes between the mitochondrial activity of different sperm subpopulations from boar ejaculates as well as in the number of spermatozoa included in each subpopulation. Similarly, other studies have shown that the distribution of spermatozoa within specific motile subpopulations is altered during in vitro capacitation [26,27], probably because some spermatozoa pass from one subpopulation to another as a result of changes in their structure and functionality in response to the capacitating effectors. In addition, an increase in the sperm subpopulation characterized by a hyperactivated-like movement was also detected during capacitation in some species [6,28].

The motility pattern of each subpopulation and the proportion of spermatozoa distributed within them can also be affected by the cryopreservation process. In several species, freezing—thawing procedures altered those motile sperm subpopulations present in fresh ejaculates [4,22,29,30,31].

All these findings highlight the relevance to study the response of different sperm subpopulations to diverse conditions or treatments, since it provides meaningful information that may otherwise be lost or masked if only mean values are considered. The combination of certain techniques of sperm evaluation, such as computer-assisted sperm analysis system (CASA) and imaging flow cytometry, allow to evaluate diverse sperm parameters simultaneously and objectively in different sperm subpopulations, ensuring a more accurate analysis [32].

Our previous studies revealed that cryopreservation of ram spermatozoa altered multiple changes that occur progressively and simultaneously during in vitro capacitation, mainly due to the detrimental effects observed on sperm quality and the reduction in the time required to attain them in comparison to fresh spermatozoa [20,33,34]. However, such results were obtained taking into account the whole sperm population, which probably contains non-responding subpopulations and subpopulations of spermatozoa with different capacitation stages.

To our knowledge, this is the first comparative study in ram spermatozoa that investigates how cryopreservation affects the subpopulation structure of motile spermatozoa throughout capacitation in comparison to fresh ones. In addition, this study also explored whether there was any relationship between the different motile sperm subpopulations and those changes associated with capacitation, since little is known about such association. We hypothesize that cryopreservation will disturb the dynamics of different sperm subpopulations during capacitation by reducing potentially those motile subpopulations implicated in the fertilization process. Moreover, it is possible that the same subpopulation of ram spermatozoa that develops a hyperactivated-like movement during capacitation also shows a marked increase in mitochondrial activity and tyrosine phosphorylation in different sperm regions, two relevant parameters that usually increase under this phenomenon.

The present study provides valuable information on the relative proportion of spermatozoa that are able to reach the capacitated state and become hyperactivated at a given time in a semen sample subjected to cryopreservation, which would improve our current understanding about how cryopreservation alters the fertilization potential of fresh ejaculates. It is well known that a greater reproductive success implies, among others, that a correct number of capacitated and hyperactivated spermatozoa must be present in a specific time interval, which in turn seems to be related with the distribution of motile sperm subpopulations during this event [27]. Therefore, the comparative study of subpopulation dynamics during in vitro capacitation in fresh and frozen-thawed samples can be critical to improve freezing—thawing procedures and even capacitating media. In this sense, this type of evaluation could be an interesting tool to assess whether the addition of new compounds to the freezing extender or modifications in the cryopreservation protocols can extend the fertilizing potential of a considerable number of sperm.

## 2. Materials and Methods

### 2.1. Samples and Cryopreservation

Four Manchega rams with an age range from 4 to 5 years were employed in the present experiment. All males were kept at the experimental farm of University of Castilla-La Mancha (UCLM) under standard nourishing and housing conditions. Semen was obtained at one-week intervals using artificial vagina by the UCLM Reproduction Biology Group which is officially authorized for collecting and storing sheep semen (ES07RS02OC), following RD 841/2011. Three ejaculates from each ram with a total motility greater than 65%, a quality of movement above 3.5 and a concentration that ranged between 3000 to 3500 × 10^6^ sperm/mL were employed in the study. The experiment was repeated three times, and ejaculates from different males (one ejaculate per male) were mixed every time to avoid individual differences (n = 12 ejaculates, 4 rams). After mixing the ejaculates, each pool was split into two portions: one was processed as fresh semen and the other as frozen-thawed semen.

Regarding the second fraction, semen was diluted in a commercial extender to 200 × 10^6^ sperm/mL (Biladyl^®^ with 20% egg yolk, Minitube; Tiefenbach, Germany) and cryopreserved following the protocol described by Peris-Frau et al. [33]. Straws were defrosted at 37 °C for 30 s in a water bath.

### 2.2. Sperm Capacitation and Experimental Design

Seminal plasma, extenders and debris were removed in fresh and frozen-thawed semen using single columns of Percoll^®^ 45% (700× *g*; 10 min). Following centrifugation, sperm pellets were diluted to 10 × 10^6^ sperm/mL in a capacitating or non-capacitating media and then incubated up to 240 min at 38.5 °C under 5% CO_2_. For non-capacitating medium (NC), which was used as negative control, polyvinyl alcohol (0.1%) was added to synthetic oviductal fluid [35], while for capacitating medium (CAP), estrous sheep serum (2%) was added to synthetic oviductal fluid [36,37,38].

Different motility parameters, mitochondrial activity, viability and protein tyrosine phosphorylation were evaluated simultaneously in fresh and frozen-thawed spermatozoa after 1, 5, 15, 30, 60, 120, 180 and 240 min under CAP conditions and at 0, 15 and 240 min under NC conditions. In all figures represented in the results section, the dots indicate the sampling time for each condition. Since the NC medium acts as a negative control, only three representative times were evaluated. We selected 0 min to assess the initial mean values of all these parameters in fresh and frozen-thawed spermatozoa before considering the effect of incubation time. The other two negative controls, 15 and 240 min NC, were selected to estimate the effect of incubation media and time, because our previous study [20] revealed that frozen-thawed spermatozoa underwent capacitation-associated changes after 15 min in CAP medium, taking place at 240 min in fresh spermatozoa. By doing so, we avoided overlapping measures and obtained a more precise evaluation of those capacitation-associated changes over time.

### 2.3. Estimation of Sperm Motility Variables Using CASA

Sperm kinematics were measured in fresh and frozen-thawed samples using a computer-aided sperm analyzer (Sperm Class Analyzer^®^ CASA System, Microptic; Barcelona, Spain). Aliquots of 5 µL sperm/sample were placed on a Makler chamber (10 µm depth; Haifa Instruments, Haifa, Israel) for each time interval and then observed in a microscope (Nikon Eclipse 80i, Tokyo, Japan) equipped with a warmed stage (37 °C) and a Basler A302fs digital camera (Basler Vision Technologies, Ahrensburg, Germany). Evaluations were made at 10× magnification and at least ten fields or 200 spermatozoa were recorded for each sample. Settings were adjusted to ram spermatozoa (20–90 µm^2^ for head area and 25 frames/s for image sampling frequency). Recorded parameters were curvilinear velocity (VCL, μm/s), straight line velocity (VSL, μm/s), average path velocity (VAP, μm/s), linearity (LIN, %), straightness (STR, %), wobble (WOB, %), lateral head displacement (ALH, μm), beat cell frequency (BCF, Hz) and total motility (%). VCL, VSL, STR, LIN, VAP, ALH, WOB and BCF were only calculated in motile spermatozoa.

### 2.4. Estimation of Mitochondrial Activity, Viability and Protein Tyrosine Phosphorylation by Flow Cytometry

Mitochondrial activity, viability and protein tyrosine phosphorylation were estimated using a FlowSight^®^ imaging flow cytometer (Amnis^®^, Merck-Millipore; Germany) controlled with the INSPIRE^®^ software (v.3). In all cases, a scatterplot with aspect ratio and area was applied to differentiate the sperm populations from debris. The aspect ratio and area threshold were set to ≤0.40 and ≥50–≤400 μm^2^, respectively, to acquire events compatible with ram sperm structure. A total of 10,000 events/sample were recorded and further analyzed.

Mitochondrial activity was determined at different times in fresh and frozen-thawed samples using a dual fluorescence staining. Briefly, sperm samples were incubated for 15 min in the dark at 37 °C in SOF-PVA-HEPES with 25 nM of YO-PRO-1 (Invitrogen, Barcelona, Spain) and 100 nM of MitoTracker Deep Red (Invitrogen, Barcelona, Spain). After incubation, all samples were evaluated with the FlowSight^®^ flow cytometer, and raw data were further analyzed with the IDEAS^®^ software to identify four different subpopulations in each sample (Figure S1): viable spermatozoa with active mitochondria (MitoTracker+/YO-PRO-1−), viable spermatozoa with inactive mitochondria (this subpopulation was practically non-existent, MitoTracker−/YO-PRO-1−), apoptotic spermatozoa with active mitochondria (MitoTracker+/YO-PRO-1+), apoptotic spermatozoa with inactive mitochondria (MitoTracker−/YO-PRO-1+). YO-PRO-1 was excited with a 488 nm laser (20 mW intensity) and collected in channel 2 (505–560 nm), while MitoTracker Deep Red was excited with a 642 nm laser (5 mW intensity) and collected in channel 11 (642–740 nm).

Viability was also measured at different times in fresh and frozen-thawed samples using a dual fluorescence staining. The staining solution contained 3 µM of PI and 25 nM of YO-PRO-1 in SOF-PVA-HEPES. Sperm samples were stained and subsequently evaluated with the FlowSight^®^ flow cytometer. Raw data were further analyzed with the IDEAS^®^ software to identify three different subpopulations in each sample (Figure S2): viable spermatozoa (YO-PRO-1−/PI−), apoptotic spermatozoa (YO-PRO-1+/PI−) and dead spermatozoa (YO-PRO-1−/PI+). YO-PRO-1 and PI were excited with a 488 nm laser (20 mW intensity) but collected in different channels, channel 2 (505–560 nm) was used for YO-PRO-1, while channel 4 (595–642 nm) for PI.

Protein tyrosine phosphorylation (pY) was evaluated during diverse time intervals in fresh and frozen-thawed samples as described by Peris-Frau et al. [33]. Briefly, 3 µM of PI was added to distinguish live spermatozoa. After that, fixation and permeabilization of multiple sperm samples was done by using paraformaldehyde in PBS (2%) for 10 min and BD FACS™ Permeabilizing Solution for 10 min (0.1%), respectively. Then, spermatozoa were washed in PBS. After 30 min at 38.5 °C with the blocking buffer (BSA at 10%), all samples were incubated with anti-phosphotyrosine monoclonal antibody Clone 4G10 (1:300 dilution in blocking buffer, Merck-Millipore; Madrid, Spain) for 1 h at 38 °C. Spermatozoa were then washed and incubated with FITC-conjugated anti-mouse IgG antibody (1:300 dilution in blocking buffer, Sigma-Aldrich; Madrid, Spain) for 1 h in the dark at 38 °C. Spermatozoa incubated with IgG1-FITC (isotype from murine myeloma, clone MOPC 21; 1:300 dilution, Sigma-Aldrich) instead of the primary antibody were used as negative control. Finally, all samples were assessed with the FlowSight^®^ imaging flow cytometer. FITC and PI were excited with a 488 nm laser (20 mW intensity) but collected in different channels, channel 2 (505–560 nm) was used for FITC, while channel 4 (595–642 nm) for PI. Raw data were further analyzed with the IDEAS^®^ as described by Matamoros-Volante et al. [39] to quantify pY fluorescence intensity in different regions of live spermatozoa in each sample. After excluding dead spermatozoa (PI+) with the histogram intensity channel 4, we selected focused and non-agglutinated live spermatozoa (single sperm) using the histogram Gradient Root Mean Square (RMS; threshold from 65 to 78) and the scatterplot with the features area and aspect ratio (area between 25 and 300 μm^2^ and aspect ratio lower than 0.4), respectively. Later, we created multiple masks to distinguish the different sperm segments (head, midpiece, principal piece and tail, which includes midpiece and principal piece) in ram sperm images using the imaging analysis tool of IDEAS^®^ software named masks. The midpiece and principal piece are different parts of the sperm flagellum involved in sperm motility, hyperactivation and energy production. The first one is located between the connecting piece or neck and the principal piece. This part of the flagellum contains the outer dense fibers and the mitochondria, which are responsible for ATP production, ROS production and modulating cell apoptosis. The principal piece is ubicated between the midpiece and the end piece of the flagellum. This part of the flagellum that contains the fibrous sheath is implicated also in sperm motility and ATP production through the metabolic pathway called glycolysis.

The masks were then used to select those images which were properly segmented. Diverse scatterplots with specific features created for each mask were used for the segmentation and gating process (Figure S3). First, we chose the sperm images with correct head segmentation using a scatterplot with head aspect ratio and head length. Spermatozoa with a head aspect ratio higher than 0.4 and a head length lower than 20 μm were gated as true head. True head population was used to create other scatterplots and select spermatozoa with correct principal piece and tail segmentation. For the principal piece (part of the flagellum located between the midpiece and the end piece that contains the fibrous sheath), we used the features of principal piece aspect ratio and principal piece length in the scatterplot (sperm with a principal piece aspect ratio lower than 0.6 and a principal piece length lower than 30 μm were gated as true principal piece), while for tail, we used the features of tail aspect ratio and tail length in the scatterplot (sperm with a tail aspect ratio lower than 0.58 and a tail length lower than 45 μm were gated as true tail). Finally, those spermatozoa with proper head and principal piece segmentation were used to select spermatozoa with a correct midpiece segmentation (sperm with a midpiece aspect ratio lower than 0.8 and a midpiece length lower than 15 μm were gated as true midpiece). The midpiece is the part of the flagellum that contains the mitochondria sheath and the outer dense fibers and is located between the connecting piece (adjacent to the sperm head) and the principal piece.

After the segmentation and gating process, we created other scatterplots with the gated populations (true head population, true principal piece population, true midpiece population, true tail population) to quantify pY in different sperm regions by estimating the mean fluorescence intensity of all the spermatozoa properly segmented in each region. All samples were normalized to the negative control of 0 min NC (the mean fluorescence intensity of each sperm region was divided by the mean fluorescence intensity of the same region at 0 min NC).

### 2.5. Statistical Evaluation

Subpopulation analysis was carried out following the two-step procedure proposed by Martinez-Pastor et al. [22] and explained in detail in Maroto-Morales et al. [40]. Sperm motile variables used to drive the sperm classification were VCL, LIN, ALH and BCF. These variables were chosen considering the relationship among all of them with a Variable Group Analysis. In order to consider how individual variation in the 4 ejaculates pooled could affect the clustering process, we proposed to carry out the clustering analysis many times using subsamples. Thus, the whole database was randomly divided into 10 subsamples and clustering analysis was carried out 10 times, excluding one of the different 10 subsamples on each time, i.e., in the first analysis, all subsamples except the first one were included; in second analysis, all subsamples except subsample 2 were included, and so on. Previous to the clustering procedure, a standardization of data was performed to avoid the negative effects of considering classification variables measured in different scales. For the standardization (also called normalization), variables were centered (mean equals to 0) and scaled (standard deviation equals to 1). The two-step procedure was carried out as follows: in a first step, spermatozoa were classified into clusters using a non-hierarchical procedure, the k-means clustering. The optimal number of non-hierarchical clusters to keep for the next step was decided based on the Silhouette Average Width criterion [41]. In a second step, centroids of clusters obtained in the first clustering step were grouped using a hierarchical procedure, obtaining the final subpopulations. Optimal number of clusters from this hierarchical procedure were decided based on the Hubert *Г* coefficient [42,43] and the L method [44].

For each of the 10 data subsets in the 10-fold sampling procedure, the proportion of spermatozoa belonging to each subpopulation was calculated, and average and standard deviation were obtained as a summary from these 10 data subsets. Differences in the abundance of different subpopulations between treatments and incubation times as well as differences in mitochondrial activity and tyrosine phosphorylation were studied using a regression model. When *p* < 0.05 was obtained, the differences were considered significant.

In addition, the correlations between the percentage of spermatozoa belonging to each subpopulation and the proportion of spermatozoa with active mitochondria or with phosphorylation in different regions were studied using Pearson’s coefficient. The correlations were calculated separately for fresh and frozen-thawed samples considering all the values obtained throughout the different incubation times under CAP or NC conditions in the three replicates. *p* < 0.01 was used to differentiate significant correlations.

## 3. Results

### 3.1. Total Sperm Motility, Mitochondrial Activity and Tyrosine Phosphorylation in Different Regions during Capacitating and Non-Capacitating Conditions in Fresh and Frozen-Thawed Spermatozoa

**Figure 1:**
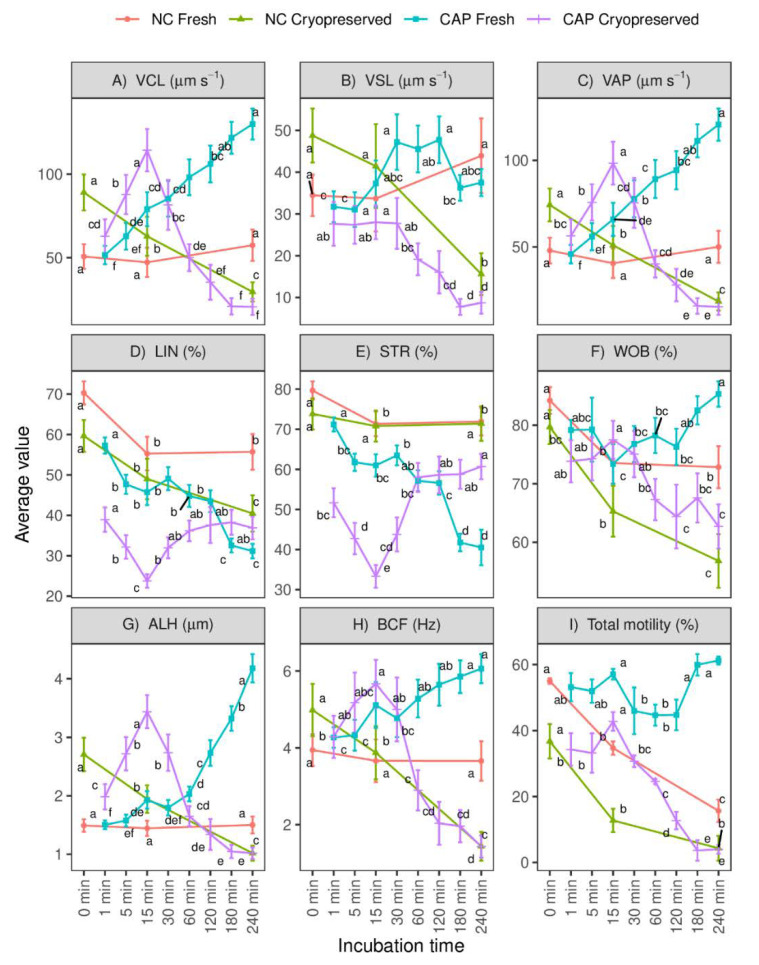
Overall motility and kinematic characteristics of fresh and frozen-thawed spermatozoa incubated in capacitating (CAP) and non-capacitating (NC) media over time. (**A**) VCL: curvilinear velocity; (**B**) VSL: straight line velocity; (**C**) VAP: average path velocity; (**D**) LIN: linearity; (**E**) STR: straightness; (**F**) WOB: wobble; (**G**) ALH: lateral head displacement; (**H**) BCF: beat cell frequency and (**I**) total motility were the parameters evaluated. Values are expressed as means ± S.E.M (n = 12 ejaculates, 4 rams × 3 replicates). Different letters denote substantial changes (*p* < 0.05) among incubation times within each type of sample.

Non-capacitating (NC) conditions progressively decreased the total motility of fresh and frozen-thawed spermatozoa throughout time (Figure 1I). However, a different response was observed between both types of samples under capacitating (CAP) conditions. While in fresh samples the percentage of motile spermatozoa presented fluctuations during CAP incubation (with major changes between 30–120 min to 180–240 min), the motility of frozen-thawed spermatozoa began to decrease in a time-dependent manner after 15 min, showing significant differences (*p* < 0.05) from 60 min (Figure 1I).

In relation to the other kinematic descriptors depicted in Figure 1A–H, most of them, especially VCL, LIN, VAP, ALH and STR, showed the greatest variations when ram spermatozoa were incubated in the CAP medium compared to NC, occurring at 180–240 min in fresh samples and at 15 min in frozen-thawed samples.

**Figure 2:**
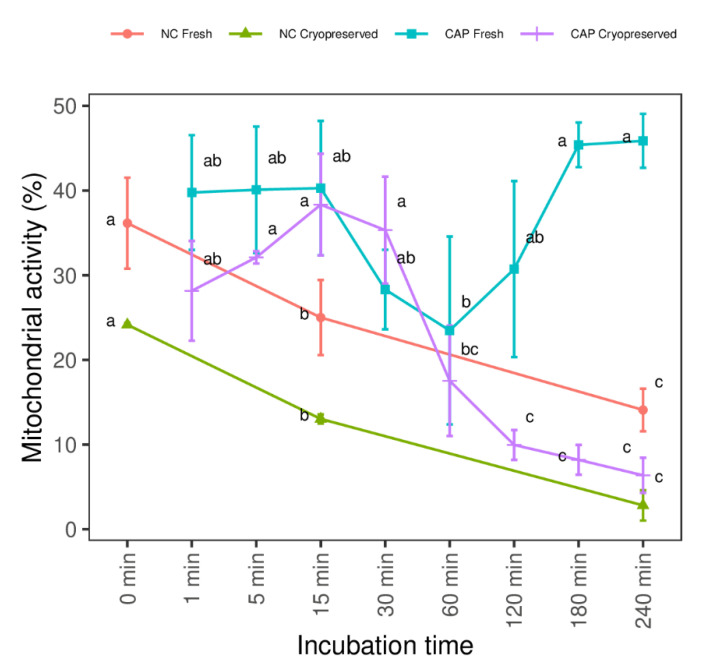
Mitochondrial activity of fresh and frozen-thawed spermatozoa incubated in capacitating (CAP) and non-capacitating (NC) media over time. Values showed the average proportion of live spermatozoa with active mitochondria (± S.E.M) in each time (n = 12 ejaculates, 4 rams × 3 replicates). Different letters denote substantial changes (*p* < 0.05) among incubation times within each type of sample.

Similar to the motility results, the proportion of viable sperm with active mitochondria decreased in a time-dependent manner during NC conditions in fresh and frozen-thawed samples. This also occurred after 30 min in CAP medium in frozen-thawed spermatozoa, while in fresh spermatozoa, the mitochondrial activity oscillated throughout CAP incubation (Figure 2).

**Figure 3:**
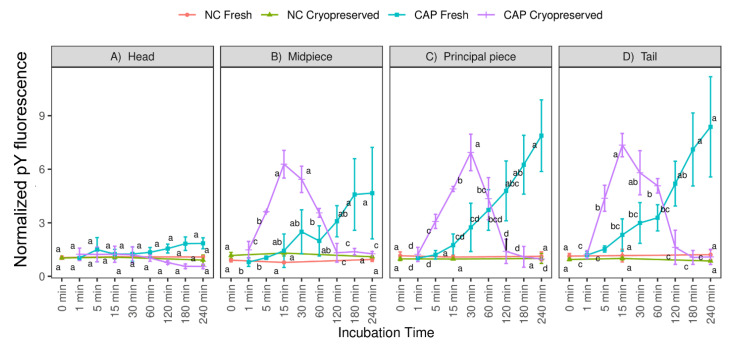
Protein tyrosine phosphorylation (pY) in different sperm regions throughout incubation of fresh and frozen-thawed ram spermatozoa in capacitating (CAP) and non-capacitating (NC) media (n = 12 ejaculates, 4 rams × 3 replicates). The sperm regions evaluated were (**A**) head (includes sperm with fluorescence in the entire head, acrosome or equatorial segment), (**B**) midpiece, (**C**) principal piece and (**D**) tail (includes spermatozoa with fluorescence in the midpiece and principal piece). The mean fluorescence intensity of all spermatozoa in each region and time was normalized to the mean fluorescence intensity of the same region at 0 min NC (±S.E.M). Different letters denote substantial changes (*p* < 0.05) among incubation times within each type of sample.

Another parameter assessed during the incubation period was the mean fluorescence intensity of tyrosine-phosphorylated proteins in different regions of live spermatozoa (Figure 3A–D). In vitro capacitation significantly increased tyrosine phosphorylation of flagellar proteins present either in the midpiece or principal piece (Figure 3B–D). The greatest values of tyrosine phosphorylation during CAP conditions were recorded at 15–30 min in the midpiece and entire flagellum (midpiece + principal piece) and at 15 min in the principal piece for frozen-thawed spermatozoa, whereas in fresh spermatozoa they were found at 180–240 min in the midpiece and at 120–240 min in the principal piece and entire flagellum. In contrast, this increment was not observed when fresh and frozen-thawed spermatozoa were incubated in NC medium (*p* > 0.05). Finally, tyrosine phosphorylation hardly changed in the sperm head of fresh and frozen-thawed samples during the entire incubation in NC (*p* > 0.05). Although no significant changes were found in head phosphorylation under CAP conditions, there is a tendency to increase in fresh samples at the end of incubation, which occurs oppositely in frozen-thawed samples (Figure 3A).

### 3.2. Characterization of Motile Sperm Subpopulations Present in Fresh and Frozen-Thawed Semen Samples during Capacitating and Non-Capacitating Conditions

Clustering analysis revealed the existence of four sperm subpopulations in fresh and frozen-thawed samples based on their kinematic characteristics. Such analysis included all the values obtained in the three replicates from all incubation times measured in both media, either for fresh or frozen-thawed spermatozoa. The variables used to classify spermatozoa were VCL, LIN, ALH and BFC. The motility pattern of each sperm subpopulation and the difference between them are described in Table 1.

Subpopulation 1 (SP1) contained spermatozoa with low velocity (lowest values of VCL, VSL and VAP) but with high progressiveness (highest values of LIN and STR). In addition, this subpopulation also had a low oscillatory movement, as shown by the lowest values of WOB, ALH and BCF.

Subpopulation 2 (SP2) included spermatozoa with moderate velocity, as shown by the second lowest values of VCL, VSL and VAP, but with high progressiveness (high LIN and STR) and low oscillatory movement (low WOB, ALH and BCF).

Subpopulation 3 (SP3) contained fast and progressive spermatozoa with a high oscillatory movement, as shown by the high values of all kinematic descriptors.

Subpopulation 4 (SP4) represented the fastest and less progressive spermatozoa with the highest oscillatory movement, as shown by the highest values of VCL, VAP, WOB, ALH and BCF and the lowest values of LIN and STR. This subpopulation could represent those spermatozoa with hyperactivated-like movement, as described previously by García-Álvarez et al. [6].

### 3.3. Variations in the Distribution of Motile Sperm Subpopulations throughout the Incubation of Fresh and Frozen-Thawed Ram Spermatozoa

**Figure 4:**
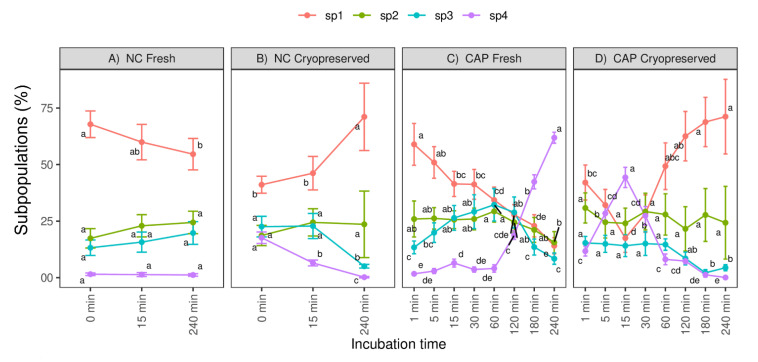
Distribution of fresh and frozen-thawed motile spermatozoa within subpopulations (SP1, SP2, SP3, SP4) over the incubation in capacitating (CAP) and non-capacitating (NC) media. (**A**) Motile subpopulations of fresh spermatozoa during NC conditions. (**B**) Motile subpopulations of frozen-thawed spermatozoa during NC conditions. (**C**) Motile subpopulations of fresh spermatozoa during CAP conditions. (**D**) Motile subpopulations of frozen-thawed spermatozoa during CAP conditions. SP1: progressive spermatozoa with low velocity; SP2: progressive spermatozoa with medium velocity and low oscillatory movement; SP3: fast and progressive spermatozoa with a high oscillatory movement; SP4: fast spermatozoa with the highest oscillatory movement and the lowest linearity (sperm with hyperactivated-like movement). Values are expressed as mean percentages ± S.E.M (n = 12 ejaculates, 4 rams × 3 replicates). Different letters denote substantial changes (*p* < 0.05) among incubation times within each type of sample.

Although the four sperm subpopulations were present in all sperm samples, their distribution changed between samples, incubation media and times (Figure 4). The proportion of motile spermatozoa included in Subpopulation 1 (SP1) decreased in a time-dependent manner during the incubation of fresh spermatozoa under CAP and NC conditions, reaching the lowest percentages at 240 min. However, in frozen-thawed spermatozoa, SP1 significantly (*p* < 0.05) decreased up to 15 min and then progressively increased throughout CAP incubation, occurring at 240 min in NC conditions. This subpopulation (SP1) also represented the main subpopulation in NC conditions compared to CAP, either in fresh or frozen-thawed spermatozoa.

The second subpopulation that underwent the most significant changes during CAP conditions was Subpopulation 4 (SP4). The number of motile spermatozoa included in SP4 increased drastically (*p* < 0.05) from 120 to 240 min in CAP conditions for fresh spermatozoa, reaching the highest percentage at the end of incubation (240 min). In frozen-thawed spermatozoa, this subpopulation (SP4) significantly (*p* < 0.05) increased from 5 to 30 min during CAP conditions, with the highest percentage at 15 min, and then progressively declined over time (Figure 4). However, this increment was not observed during the NC incubation in fresh or frozen-thawed spermatozoa. In fresh spermatozoa, the proportion of spermatozoa belonging to SP4 was quite scarce but similar during the entire incubation under NC conditions, whereas in frozen-thawed spermatozoa, SP4 was relatively low and significantly decreased (*p* < 0.05) over time during NC conditions.

Contrary to the other subpopulations, the proportion of motile spermatozoa assigned to Subpopulation 2 (SP2) barely changed (*p* > 0.05) throughout CAP and NC conditions in fresh and frozen-thawed sperm samples; only at 240 min, this subpopulation (SP2) slightly decreased in fresh spermatozoa (Figure 4).

Finally, the percentage of motile spermatozoa included in Subpopulation 3 (SP3) was higher from 5 to 120 min in fresh spermatozoa incubated under CAP conditions, while in frozen-thawed spermatozoa, SP3 remained constant up to 60 min and then diminished at 120, 180 and 240 min of CAP conditions (Figure 4). Under NC conditions, this subpopulation (SP3) only changed by the end of incubation (240 min) in frozen-thawed spermatozoa.

### 3.4. Relationships between Motile Sperm Subpopulations, Mitochondrial Activity and Tyrosine Phosphorylation in Fresh and Frozen-Thawed Semen Samples

Different correlations were obtained in fresh and frozen-thawed spermatozoa during CAP conditions when all incubation times were considered (Table 2), but no correlations were found under NC conditions (*p* > 0.01), neither for fresh nor frozen-thawed spermatozoa.

Under CAP conditions, a negative correlation (*p* < 0.01) was found between SP1 and the different phosphorylated sperm regions (head, midpiece, principal piece and tail) in fresh spermatozoa. Besides these correlations, another negative correlation (*p* < 0.01) was found between SP1 and mitochondrial activity for frozen-thawed spermatozoa. In contrast, SP4 was positively correlated (*p* < 0.01) with mitochondrial activity and the different phosphorylated sperm regions in both types of samples (fresh and frozen-thawed spermatozoa) during CAP conditions. Regarding SP2, this subpopulation showed a negative correlation (*p* < 0.01) with the different phosphorylated sperm regions in fresh spermatozoa incubated under CAP conditions, but no correlations (*p* > 0.01) were found in frozen-thawed spermatozoa. In fresh spermatozoa, SP3 showed a positive correlation with mitochondrial activity (*p* < 0.01) under CAP conditions. Such correlation together with another positive correlation between SP3 and tyrosine phosphorylation in sperm head were also found in frozen-thawed spermatozoa during CAP conditions. The strength of association between the different SPs and the variables mentioned was mostly moderate or strong and is illustrated in Table 2.

## 4. Discussion

Our results revealed the existence of four sperm subpopulations with different swimming velocity and progressiveness in fresh and frozen-thawed ram semen subjected to the specific capacitating (CAP) and non-capacitating (NC) conditions mentioned above. Previous studies also described the presence of different motile sperm subpopulations in ram ejaculates [45], although the exact number of subpopulations differed in some cases [2,6]. These discrepancies could be attributed to differences between the multivariate clustering analysis, sperm sources or experimental conditions.

Another important finding, which was previously reported in other species [6,8,26,27,28], was the different distribution of the motile sperm subpopulations over the incubation period under CAP conditions, either in fresh or frozen-thawed samples. In earlier studies, the response of different sperm subpopulations to in vitro capacitation was only investigated in fresh or frozen-thawed semen. The present study demonstrates, for the first time, that the distribution pattern of different motile sperm subpopulations differed between fresh and frozen-thawed samples throughout in vitro capacitation. It is known that cryopreservation may alter the motility patterns of the surviving spermatozoa by changing the percentage included in each subpopulation, and even their kinematic characteristics [4,22,29,31,46]. In fact, besides altering the motility pattern of different sperm subpopulations, cryopreservation can also induce functional [6], structural [5], and molecular changes [47] in these subpopulations, which would explain their different response to the capacitating stimulus compared to the sperm subpopulations of fresh samples in our study.

Among the different sperm subpopulations identified in the present manuscript, SP1 and SP4 were the subpopulations that showed the most significant changes in fresh or frozen-thawed samples during the incubation period. In fresh semen, the percentage of spermatozoa included in SP1 decreased in a time-dependent manner during CAP incubation. The reduction in this subpopulation of slow spermatozoa during in vitro capacitation was concomitant to the gradual increase in SP4, characterized by the most rapid spermatozoa with the lowest linearity and the highest oscillatory movement. A similar situation was found in frozen-thawed semen samples until 15 min under CAP conditions. Thus, SP1 might initially contain inactive spermatozoa with a lower metabolic activity that could be activated in the presence of capacitating factors or in the female reproductive tract at a specific time interval as have been suggested by Muiño et al. [48]. Once these spermatozoa have been activated, they would pass from SP1 to SP4.

However, in frozen-thawed samples, not only the proportion of spermatozoa included in SP1 decreased during capacitation. Prolonged incubations (60 min or more) led to the increment of SP1. Such increment can be the result of cell deterioration in frozen-thawed samples, which clearly affects the quality of sperm movement. This hypothesis is further supported by the higher proportion of dead spermatozoa found in frozen-thawed samples after 60 min and by the negative correlation found between SP1 and mitochondrial activity in frozen-thawed spermatozoa as well as by the gradual reduction in mitochondrial activity in these samples. Capacitation is a transitory event that can lead to cell death if capacitated spermatozoa do not fertilize the oocyte at a given time [23,49]. This event seems to take place faster after cryopreservation, around 15–30 min in ram spermatozoa [20], which considerably reduces the lifespan of spermatozoa. In our study, the increment of SP1 in frozen-thawed samples occurred simultaneously with the reduction in SP3 and SP4 during in vitro capacitation. In this sense, SP1 may initially include in frozen-thawed samples those inactive spermatozoa, and after a prolonged incubation, those fast or capacitated and hyperactivated-like spermatozoa originally present in SP3 and SP4, which are no longer functional because they are in an early stage of metabolic damage and cell deterioration.

SP2 represented another motile subpopulation found in our study that was formed by those moderately slow spermatozoa but progressive with a low oscillatory movement. This subpopulation can be considered as non-responding, since the percentage of fresh and frozen-thawed spermatozoa belonging to SP2 remained constant throughout in vitro capacitation.

Regarding the other motile subpopulations identified, SP3 was constituted by fast and progressive spermatozoa with high linearity and oscillatory movement, while SP4 included those spermatozoa compatible with the hyperactivated-like subpopulation mentioned by other authors [6,45,50]. However, the thresholds established by these authors to classify spermatozoa as hyperactivated differed from ours, probably due to differences in the incubation media and image sampling frequency [51].

When the proportion of fresh and frozen-thawed spermatozoa assigned to SP3 and SP4 was investigated through the incubation time, a different response was observed between both type of samples. In fresh semen samples, the percentage of spermatozoa showing rapid and progressive movement (SP3) significantly increased until 60 min under CAP conditions and then began to decline concomitantly with the increment of those spermatozoa showing rapid, curvilinear and high oscillatory movement (SP4). These results, together with the absence of such changes in SP3 during NC conditions, suggest that SP3 could represent, as has been earlier reported [28], those spermatozoa that would be starting to develop a hyperactivated-like pattern, shifting from progressive to vigorous and asymmetrical flagellar movement, at least in fresh samples. This increment in SP3 was not observed in frozen-thawed samples during CAP conditions, which clearly indicates that cryopreservation affected the spermatozoa included in SP3 in such a way that they appeared not to respond to sperm capacitation.

Our results also demonstrated that only in vitro capacitation increased the proportion of fresh and frozen-thawed spermatozoa, showing a fast, non-linear and high oscillatory movement (SP4) occurring in frozen-thawed samples from 5 to 30 min and later in fresh samples (180–240 min). Previous studies also found that a subpopulation with similar motility characteristics increased during capacitating conditions [6,28]. Such increment was not detected under NC conditions in our experiment, and for this reason and based on the motility pattern of SP4, this subpopulation might represent those hyperactivated-like spermatozoa.

At the time intervals mentioned, the mean values of most kinematic descriptors exhibited the greatest changes. Mitochondrial activity showed an increasing trend, and the intensity of tyrosine phosphorylation was highest in the midpiece, principal piece and the whole flagellum. Thus, the main subpopulation responsible for most of the changes observed in the mean results during in vitro capacitation was SP4. In addition, this subpopulation was strongly correlated with mitochondrial activity and all the sperm regions that showed tyrosine phosphorylation during CAP conditions either in fresh or frozen-thawed samples. Therefore, SP4 could be the main subpopulation implicated in the fertilization process since it seems to contain capacitated and hyperactivated-like spermatozoa.

During capacitation, more energy is required for the functional and molecular modifications that take place, particularly those changes in motility and tyrosine phosphorylation [14,52]. In ram spermatozoa, the metabolic pathways present in mitochondria are important sources of ATP [53], explaining the positive correlations found in our experiment between mitochondrial activity and those fresh and frozen-thawed spermatozoa showing rapid-progressive movement (SP3) and hyperactivated-like motility (SP4) during in vitro capacitation.

One of the hallmarks of sperm capacitation is the overall increase in protein tyrosine phosphorylation [17,19]. Earlier studies demonstrated that the increment of tyrosine phosphorylation in spermatozoa during capacitation, especially in flagellar proteins, is strongly associated with the acquisition of hyperactivated-like motility, with both events occurring simultaneously [54,55,56,57]. Such findings agree with our results. In fact, based on these observations and our findings, tyrosine phosphorylation of flagellar proteins seems to be crucial for the transition of progressive motility (SP3) to hyperactivated (SP4).

The last interesting outcome of this study was the greater proportion of SP4 at 0 min in frozen-thawed samples compared to fresh ones. It is widely known that cryopreservation induces capacitation-like changes in a proportion of surviving spermatozoa [58]. In these spermatozoa, the selective permeability of the plasma membrane is altered, which increases calcium influx, an ion that play a major role in regulating hyperactivation [11]. This would explain the higher presence of spermatozoa showing premature hyperactivated-like kinematic characteristics at 0 min in our experiment. Similarly, earlier studies also found that the cooling step significantly increased the proportion of spermatozoa with a hyperactivated-like movement [29,48].

## 5. Conclusions

In summary, the four subpopulations of motile spermatozoa identified in fresh and frozen-thawed ram semen samples exhibited a different distribution during in vitro capacitation. Such distribution also changed between both types of samples and incubation media. In fact, the subpopulation with the fast, non-linear and the highest oscillatory movement, SP4, only increased concomitantly with mitochondrial activity and all phosphorylated sperm regions during CAP conditions, but not in NC, occurring in frozen-thawed spermatozoa faster than fresh spermatozoa. Based on these results and the positive correlations found, SP4 probably represents the subpopulation of hyperactivated-like and capacitated spermatozoa. After cryopreservation, this subpopulation (SP4) was the most affected by prolonged incubations, which indicates that the fertilization potential of frozen-thawed ram semen could be relatively short. These findings highlight the need to adapt current assisted reproductive techniques in ovine when frozen-thawed semen is used to maximize the reproductive success. In addition, this type of classification and analysis could be useful to evaluate whether the improvements of cryopreservation protocols may extend the lifespan of those spermatozoa involved in the fertilization process and even for the development of new methods to select those spermatozoa with better fertility for the reproductive biotechnologies.

## Figures and Tables

**Table 1:** Mean kinematic characteristics of the four motile subpopulations (SP) identified in fresh and frozen-thawed ram spermatozoa considering all incubation times in CAP and NC media (n = 12 ejaculates, 4 rams × 3 replicates).

Kinematic Parameters	Subpopulations			
	SP1	SP2	SP3	SP4
VCL (µm/s)	26.38 ± 1.77 ^c^	75.78 ± 1.85 ^b^	162.12 ± 1.26 ^a^	183.64 ± 3.50 ^a^
VSL (µm/s)	15.35 ± 1.85 ^b^	46.45 ± 1.14 ^ab^	87.91 ± 9.55 ^c^	54.22 ± 1.05 ^a^
VAP (µm/s)	21.32 ± 2.09 ^c^	66.49 ± 1.88 ^b^	151.93 ± 1.64 ^a^	163.15 ± 3.34 ^a^
LIN (%)	56.45 ± 3.02 ^a^	44.04 ± 4.56 ^a^	52.75 ± 3.01 ^a^	29.49 ± 0.16 ^b^
STR (%)	75.44 ± 1.22 ^a^	59.79 ± 1.74 ^a^	58.55 ± 1.84 ^a^	35.43 ± 0.26 ^b^
WOB (%)	72.78 ± 2.91 ^b^	74.31 ± 5.78 ^a,b^	87.00 ± 0.29 ^a^	90.18 ± 2.63 ^a^
ALH (µm)	1.23 ± 0.02 ^d^	2.02 ± 0.15 ^c^	2.91 ± 0.08 ^b^	5.42 ± 0.08 ^a^
BCF (Hz)	2.56 ± 0.35 ^b^	5.73 ± 0.88 ^a^	7.26 ± 0.11 ^a^	7.83 ± 0.66 ^a^

VCL: curvilinear velocity; VSL: straight line velocity; VAP: average path velocity; LIN: linearity; STR: straightness; WOB: wobble; ALH: lateral head displacement; BCF: beat cell frequency. Data are expressed as means ± S.E.M. Different letters indicate substantial changes between subpopulations (*p* < 0.05).

**Table 2:** Correlation coefficients between the percentage of motile sperm subpopulations (SP), spermatozoa with active mitochondria (mitochondrial activity) or with tyrosine phosphorylation (pY) during capacitating (CAP) conditions. Correlations were estimated separately in fresh and frozen-thawed spermatozoa considering all incubation times in CAP conditions in the three replicates.

CAP Conditions	Fresh Sperm				Frozen-Thawed Sperm			
	SP1	SP2	SP3	SP4	SP1	SP2	SP3	SP4
Mitochondrial activity	−0.18	−0.60	0.83 *	0.79 *	−0.99 *	−0.24	0.63 *	0.91 *
pY head	−0.84 *	−0.90 *	0.29	0.88 *	−0.93 *	−0.12	0.59 *	0.79 *
pY midpiece	−0.93 *	−0.91 *	0.21	0.91 *	−0.84 *	−0.56	0.40	0.90 *
pY principal piece	−0.97 *	−0.92 *	0.21	0.93 *	−0.69 *	−0.38	0.64	0.69 *
pY tail	−0.96 *	−0.95 *	0.26	0.96 *	−0.81 *	−0.60	0.51	0.86 *

* *p* < 0.01. SP1: spermatozoa with low velocity, but with high progressiveness; SP2: spermatozoa with medium velocity, but with high progressiveness; SP3: fast and progressive spermatozoa with a high oscillatory movement and SP4: fastest and less progressive spermatozoa with the highest oscillatory movement; mitochondrial activity: proportion of viable spermatozoa with active mitochondria; pY head: normalized fluorescence intensity of the head region; pY midpiece: normalized fluorescence intensity of the midpiece region; pY principal piece: normalized fluorescence intensity of the principal piece region; pY tail: normalized fluorescence intensity of the midpiece and principal piece.

## Data Availability

https://doi.org/10.5061/dryad.f7m0cfxxp.

## Supplementary Materials

The following are available online at https://www.mdpi.com/article/10.3390/biology10111213/s1, Figure S1: Flow cytometry analysis of mitochondrial activity, Figure S2: Sperm viability of fresh and cryopreserved samples during the incubation period, Figure S3: Flow cytometry analysis of different sperm regions showing tyrosine phosphorylation.
