# Supplementary material for: Impact of Cryopreservation on Motile Subpopulations and Tyrosine-Phosphorylated Regions of Ram Spermatozoa during Capacitating Conditions
Source: Biology (Basel). 2021 Nov 20;10(11):1213. doi: 10.3390/biology10111213 (PMC8614982; doi:10.3390/biology10111213)
Supplement: Supplementary file 1 [file biology-10-01213-s001.zip › Supplementary figure S1.pdf]

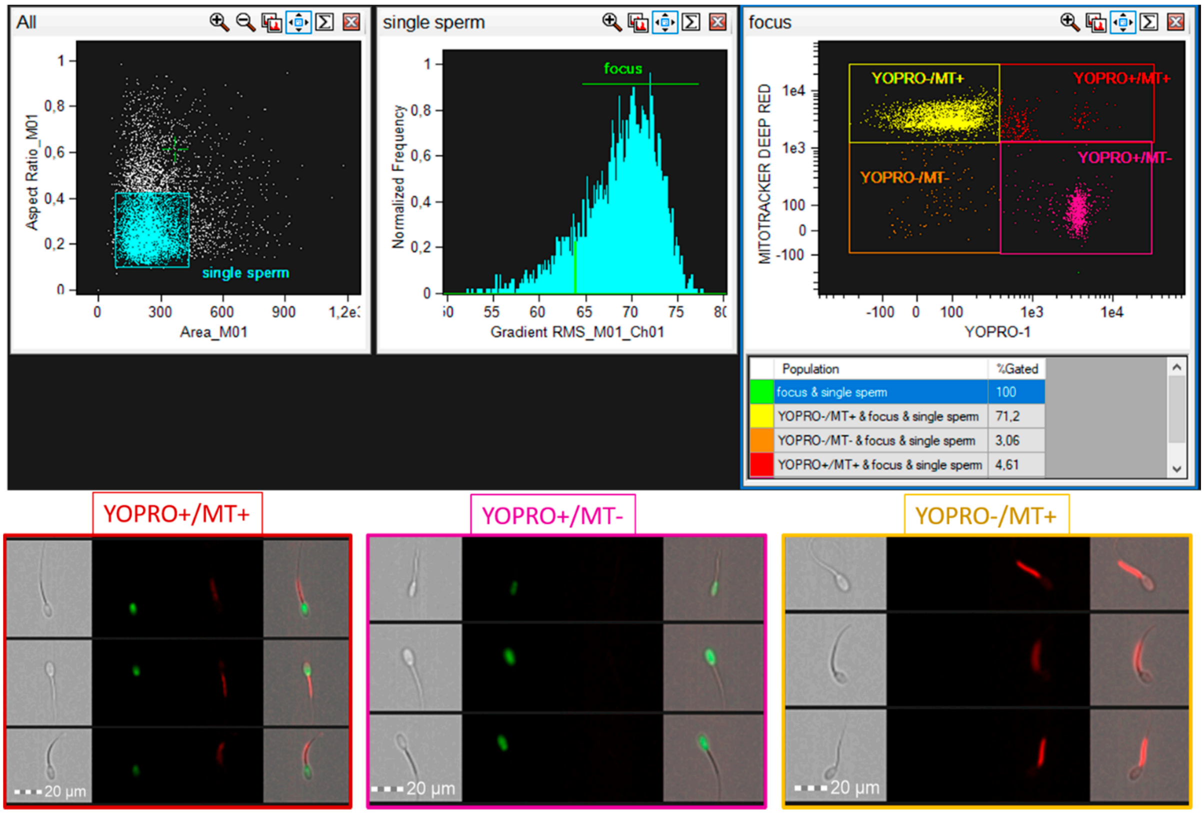

Supplementary figure S1. Flow cytometry analysis of mitochondrial activity. Different dot-plots were used to identify single sperm, focused sperm and different sperm populations (upper panels). Lower panels show images of apoptotic spermatozoa with active mitochondria (YOPRO+/MT+) apoptotic spermatozoa with inactive mitochondria (YOPRO+/MT−) and viable spermatozoa with active mitochondria (YOPRO−/MT+).
