# Supplementary material for: Impact of Cryopreservation on Motile Subpopulations and Tyrosine-Phosphorylated Regions of Ram Spermatozoa during Capacitating Conditions
Source: Biology (Basel). 2021 Nov 20;10(11):1213. doi: 10.3390/biology10111213 (PMC8614982; doi:10.3390/biology10111213)
Supplement: Supplementary file 1 [file biology-10-01213-s001.zip › Supplementary figure S2.pdf]

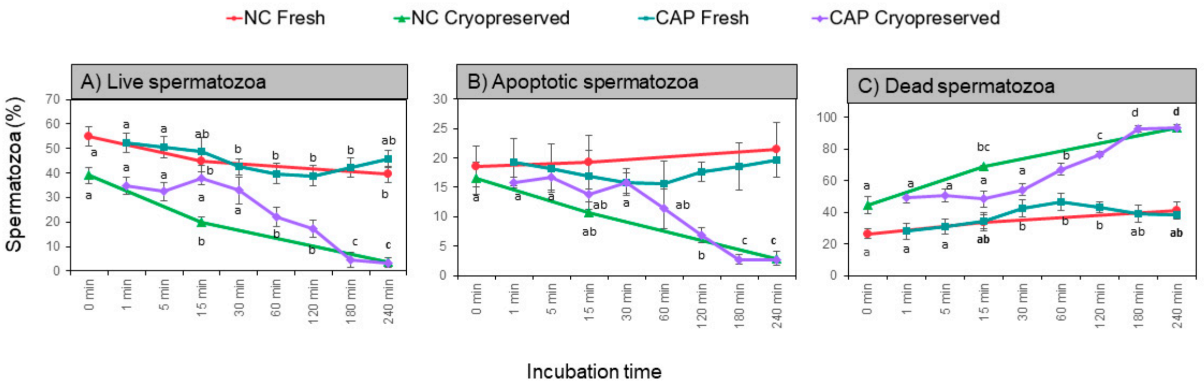

Supplementary figure S2. Sperm viability of fresh and cryopreserved samples during the incubation period under capacitating (CAP) and non-capacitating (NC) conditions. Values showed the average proportion (± S.E.M) of (A) live, (B) apoptotic and (C) dead spermatozoa in each time (n = 12 ejaculates, 4 rams). Different letters indicate significant differences ($P < 0.05$) among incubation times within each type of sample.
