# Supplementary material for: Impact of Cryopreservation on Motile Subpopulations and Tyrosine-Phosphorylated Regions of Ram Spermatozoa during Capacitating Conditions
Source: Biology (Basel). 2021 Nov 20;10(11):1213. doi: 10.3390/biology10111213 (PMC8614982; doi:10.3390/biology10111213)
Supplement: Supplementary file 1 [file biology-10-01213-s001.zip › Supplementary figure S3.pdf]

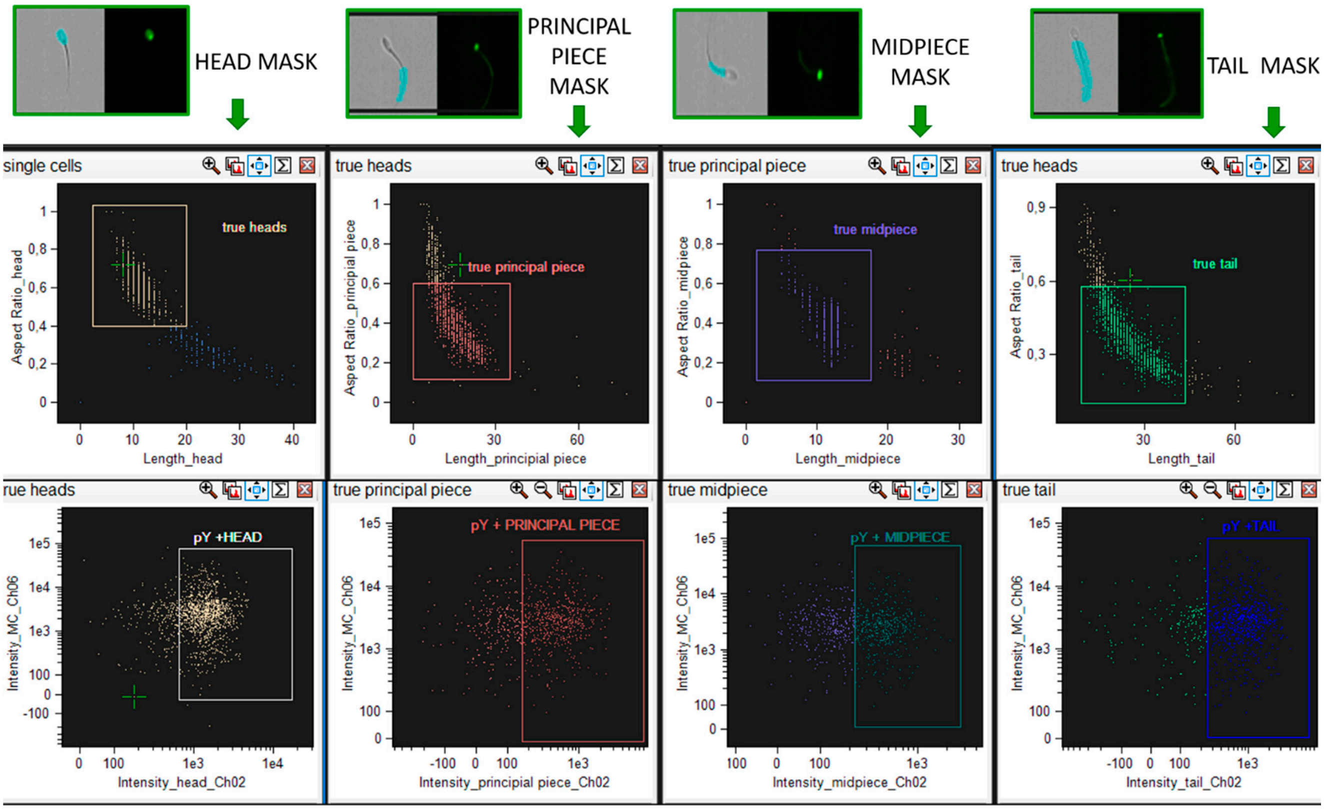

Supplementary figure S3. Flow cytometry analysis of different sperm regions showing tyrosine phosphorylation (pY). Masks were created to cover each region of ram sperm images (upper panels). Diverse dot-plots with specific features created for each mask were then used to select those populations properly segmented (middle panels) and analyze the mean fluorescence intensity of all spermatozoa in each region (lower panels).
